# Exploring predictive biomarkers of efficacy and survival with nivolumab treatment for unresectable/recurrent esophageal squamous cell carcinoma

**DOI:** 10.1007/s10388-025-01120-z

**Published:** 2025-04-24

**Authors:** Shigeto Nakai, Tomoki Makino, Kota Momose, Kotaro Yamashita, Koji Tanaka, Hiroshi Miyata, Sachiko Yamamoto, Masaaki Motoori, Yutaka Kimura, Ryohei Kawabata, Motohiro Hirao, Jin Matsuyama, Yusuke Akamaru, Hitomi Morihara, Azumi Ueyama, Yukinori Kurokawa, Eiichi Morii, Hisashi Wada, Hidetoshi Eguchi, Yuichiro Doki

**Affiliations:** 1 Department of Gastroenterological Surgery, Osaka University Graduate School of Medicine, 2-2-E2, Yamada-oka, Suita, Osaka 565-0871 Japan; 2 Department of Gastroenterological Surgery, Osaka International Cancer Institute, Osaka, Japan; 3 Department of Gastrointestinal Oncology, Osaka International Cancer Institute, Osaka, Japan; 4 Department of Surgery, Osaka General Medical Center, Osaka, Japan; 5 Department of Gastroenterological Surgery, Kindai University Nara Hospital, Nara, Japan; 6 Department of Surgery, Sakai City Medical Center, Osaka, Japan; 7 Department of Surgery, NHO Osaka National Hospital, Osaka, Japan; 8 Department of Gastroenterological Surgery, Higashiosaka City Medical Center, Osaka, Japan; 9 Department of Surgery, Osaka Rosai Hospital, Osaka, Japan; 10 Department of Clinical Research in Tumor Immunology, Graduate School of Medicine, Osaka University, Osaka, Japan; 11 Department of Pathology, Osaka University Graduate School of Medicine, Osaka, Japan

**Keywords:** Esophageal squamous cell carcinoma, Anti-PD-1 antibody, Biomarker, Tumor-infiltrating T lymphocytes, Tertiary lymphoid structure

## Abstract

**Background:**

Programmed cell death protein-1 (PD-1) blockade has improved survival for patients with esophageal squamous cell carcinoma (ESCC), but response rates are low. Biomarkers to predict who will benefit from PD-1 blockade are urgently needed.

**Methods:**

This multicenter study involved 250 patients with recurrent/unresectable advanced ESCC receiving nivolumab as second- or later-line therapy. We assessed tumor-infiltrating T lymphocytes (TILs) and tertiary lymphoid structure (TLS) density using immunohistochemistry and hematoxylin/eosin staining in surgical specimens and pre-nivolumab endoscopic biopsies.

**Results:**

In surgical specimens, clinical response (vs. non-response) to nivolumab correlated significantly with CD8^+^ lymphocyte count (160 vs*.* 95.2 cells/field, *P* = 0.0494), CD8/Foxp3 ratio (6.52 vs*.* 2.72, *P* = 0.0053), and TLS density (0.21/mm^2^ vs. 0.10/mm^2^, *P* = 0.0005). In terms of overall survival, multivariate analysis identified CD8/Foxp3 ratio (hazard ratio [HR] = 1.83, *P* = 0.0050) and TLS density (HR = 1.67, *P* = 0.0171 as independent prognostic parameters in surgical specimens. Similarly, in endoscopic biopsies, clinical response (vs. non-response) to nivolumab correlated significantly with CD8^+^ counts (254 cells/mm^2^ vs*.* 124 cells/mm^2^, *P* = 0.0344), CCR8^+^ lymphocyte count (62.6 cells/mm^2^ vs*.* 140 cells/mm^2^, *P* = 0.0355), CD8/Foxp3 ratio (2.09 vs*.* 0.89, *P* = 0.040), and CD8/CCR8 ratio (2.34 vs*.* 0.89, *P* = 0.0020). Multivariate analysis also identified CD8/CCR8 ratio in endoscopic biopsies (HR = 1.66, *P* = 0.0313) as an independent prognostic parameter.

**Conclusions:**

CD8^+^ and CCR8^+^ cell counts, CD8/Foxp3 and CD8/CCR8 ratios, and TLS density may be predictive biomarkers of therapeutic efficacy and survival with PD-1 blockade for ESCC.

**Supplementary Information:**

The online version contains supplementary material available at 10.1007/s10388-025-01120-z.

## Introduction

Esophageal cancer ranks as the sixth leading cause of cancer death globally [1, 2]. Esophageal squamous cell carcinoma (ESCC) is the predominant subtype, comprising about 90% of cases [3, 4]. Many ESCC cases are unresectable at diagnosis, and over half of those experiencing curative resection relapse [5–13]. Prognosis is grim for patients with unresectable or metastatic ESCC, with a median overall survival of 8–10 months, underscoring the urgent need for new treatments [13].

The programmed cell death protein 1 (PD-1) pathway is crucial for regulating host defenses to eradicate tumors and is implicated in tumor immune evasion. PD-1 inhibitors enhance T cell antitumor activity by blocking the PD-1 receptor-ligand interaction [14, 15]. Nivolumab, a PD-1 immune checkpoint inhibitor (ICI), showed superiority over taxane for unresectable advanced or recurrent ESCC in the ATTRACTION-3 trial [16]. However, only about 20% of patients treated with the PD-1 monoclonal antibody responded (complete and partial responses) [16], and thus biomarkers that predict who will benefit from PD-1 blockade are warranted.

The development of ICIs for various cancer types underscores the significance of the tumor immune microenvironment [17, 18]. Recently, analyses of tumor-related factors, including microsatellite instability, PD-L1 expression, and tumor-infiltrating T lymphocytes (TILs), have been conducted to identify biomarkers predicting ICI response [19]. TILs, which include cytotoxic T lymphocytes and regulatory T cells, have been linked to treatment response and prognosis with ICI therapy in multiple cancers [20–22]. We previously showed that tertiary lymphoid structure (TLS) density impacts nivolumab efficacy and survival in patients with unresectable advanced or recurrent ESCC [23]. PD-L1 expression is currently the sole biomarker for nivolumab efficacy against ESCC in clinical settings.

In this study, we aimed to evaluate candidate biomarkers for nivolumab response and prognosis in patients with unresectable/recurrent ESCC. We utilized hematoxylin/eosin (H&E) staining and immunohistochemistry (IHC) to assess CD3, CD8, Foxp3, and CCR8 expression in TILs, along with TLS density in pre-treatment samples. To our knowledge, this multi-center study is the largest biomarker study of anti-PD-1 antibody in unresectable/recurrent ESCC treated with nivolumab.

## Material and methods

### Patients

This study included 250 patients with unresectable/recurrent ESCC treated with nivolumab as second-line or later therapy from 2014 to 2022 at 15 institutions of the Clinical Study Group of Osaka University, Upper Gastrointestinal Surgery Group. Those with primary sites sampled and tumor cells left behind were included. Eligibility criteria were a histological diagnosis of ESCC refractory or intolerant to one or more prior chemotherapy regimens, and age ≥ 20 years. Patients previously treated with an ICI other than nivolumab were ineligible. Patients with a synchronous or metachronous (within 5 years) malignancy other than carcinoma in situ or mucosal carcinoma at the start of nivolumab treatment were excluded. Included patients gave written informed consent before enrollment, which was not required for patients who had died or been lost to follow-up. The study was approved by the institutional review boards of all participating institutions (no. 19146-5). This study is registered with UMIN Clinical Trials Registry, number UMIN000040462.

### Evaluations of tumor response

Although a follow-up schedule was not specified in this study, most patients had efficacy evaluations every 6–8 weeks. Tumor response was assessed using RECIST v1.1 criteria. A minimum interval of 6 weeks between measurements was needed to determine complete response (CR), partial response (PR), and stable disease (SD) [24]. Response rate was evaluated only in patients with measurable lesions. “Response” was defined as the proportion of patients achieving the best overall CR or PR.

### IHC for CD3, CD8, Foxp3, and CCR8

Pre-treatment tumor tissue samples were obtained from endoscopic biopsy at diagnosis or surgically resected specimens. In unresectable cases, analysis was performed using pre-treatment biopsy specimens. For recurrent esophageal cancer, immunohistochemical analysis was conducted using specimens from the initial surgery. Therefore, there were no overlapping cases between biopsy and surgical specimens. Specimens were fixed in 10% neutral buffered formalin for 24–72 h and embedded in paraffin. Pathologists, unaware of clinical data, selected FFPE tissues containing the deepest part of the tumor, and these samples were sent to the study center. FFPE samples were cut into 4-μm sections for IHC staining, performed using the Dako Autostainer Link 48 (Agilent Technologies, Santa Clara, CA, USA) following the manufacturer's instructions. Primary antibodies were incubated for 30 min at room temperature. The specificities of the monoclonal antibodies used for IHC staining were confirmed with human tonsil tissue sections as positive control. Primary antibodies were monoclonal antibodies for CD3 (SP7, 1:200; Abcam, Cambridge, UK), CD8 (C8/144B, 1:100; Agilent Technologies, Santa Clara, CA, USA), Foxp3 (236A/E7, 1:1600; Abcam, Cambridge, UK), and CCR8 (433H, 1:10,000, BD Biosciences, Oxford, UK).

### Evaluations of CD3, CD8, Foxp3, and CCR8 in endoscopic biopsy and surgical specimens

**Fig. 1:**
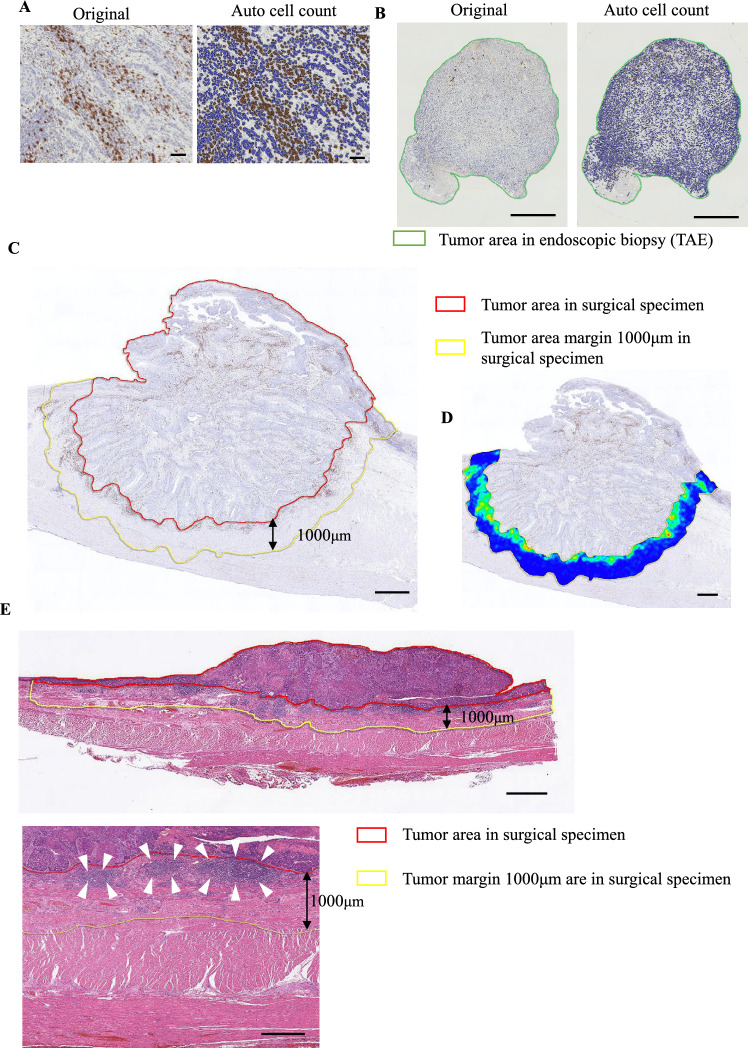
Evaluations of CD3^+^, CD8^+^, Foxp3^+^, and CCR8^+^ lymphocyte counts and TLS density in endoscopic biopsy and surgical specimens. **A** Auto count of CD3^+^, CD8^+^, Foxp3^+^, and CCR8^+^ lymphocytes using HALO software (Indica Labs, Corrales, NM, USA). **B** Representative slide of CD8 immunostaining of an endoscopic biopsy and auto count of CD8^+^ lymphocytes. The total number of TILs, including CD3^+^, CD8^+^, Foxp3^+^, and CCR8^+^ lymphocytes, was automatically counted using HALO software and divided by the sum of all biopsy tumor areas, defined as the total number of positive cells counts per tumor area in the endoscopic biopsy (the area inside the green line) (/mm^2^). **C** The tumor area margin of 1000 μm in a surgical specimen (the area inside the yellow line), defined as the area 1000 µm outward from the boundary between the tumor tissue (the area inside the red line). **D** A heatmap created using HALO software; the top five TIL “hotspots” (5 tiles with the most TILs) were selected based on a heatmap of the tumor area margin of 1000 μm in a surgical specimen. **E** The tumor area margin of 1000 μm in a surgical specimen (the area inside the yellow line), defined as the area 1000 µm outward from the boundary between normal tissue and tumor tissue (the area inside the red line). We defined TLS density by calculating the number of TLSs per millimeter in H&E-stained sections. Scale bars: **A** = 100 µm, **B** = 500 µm, **C** = 2 mm, **D** = 1 mm, **E** = 2 mm (top), 1 mm (bottom)

Every stained slide was scanned with a Ventana iScan HT (Roche Diagnostics, Sant Cugat, Spain) to obtain 20 × digital images. Two independent observers (S.N. and T.M.) assessed all slides to define the tumor core, blinded to clinicopathological data. Disagreements were resolved by conference. Whole slide images were analyzed using HALO software (Indica Labs, Corrales, NM, USA) to identify and quantify stained cells (Fig. 1A).

For endoscopic biopsy samples, HALO software automatically counted TILs, including CD3^+^ , CD8^+^ , Foxp3^+^ , and CCR8^+^ lymphocytes, and divided by the total biopsy tumor area (mm2) to get positive cell counts per tumor area (Fig. 1B). The median value was used to categorize two groups. Correlations between these counts and the response to ICI and patient prognosis were evaluated. Ratios of CD8^+^ to Foxp3^+^ and CD8^+^ to CCR8^+^ lymphocytes were also calculated and evaluated similarly.

**Fig. 2:**
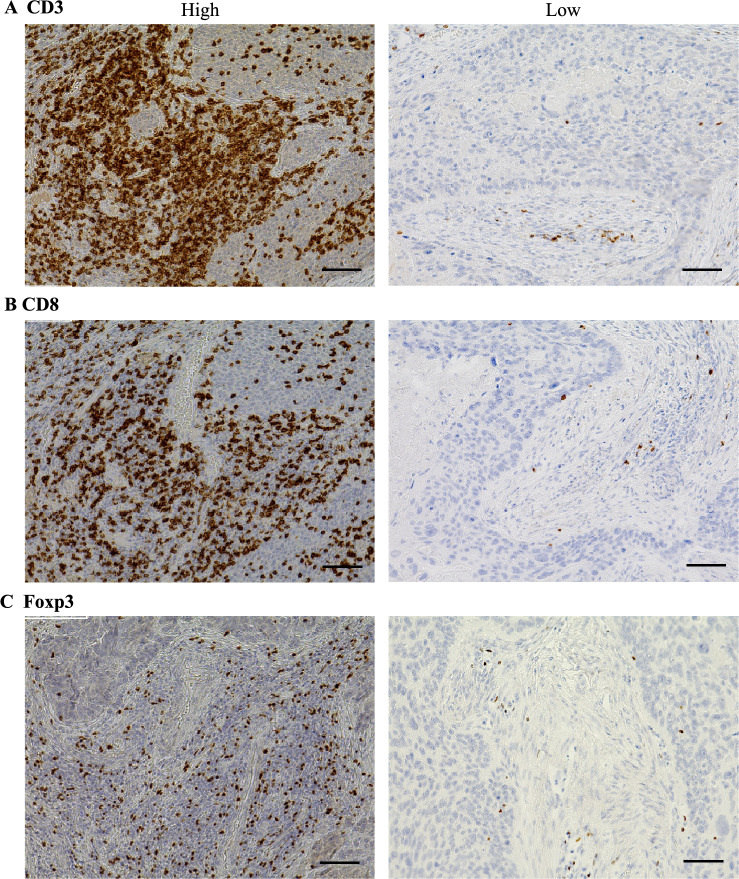
Representative slides of low and high staining densities for CD3^+^, CD8^+^, and Foxp3^+^ lymphocytes in a surgical specimen. **A** CD3; **B** CD8; **C** Foxp3. Scale bars: 100 µm

Each surgical specimen was delineated by a 1000 μm margin from the boundary between normal and tumor tissue (Fig. 1C). A heatmap using HALO software selected the top five TIL “hotspots” in surgical specimens (Fig. 1D). The number of TILs was automatically counted at 200 × magnification, and the mean of the five hotspots was calculated (Supplementary Fig. S1). The median value was the cutoff for categorizing samples. Positive control (tonsil) and representative slides of low or high densities of CD3^+^, CD8^+^, Foxp3^+^, and CCR8^+^ lymphocytes were provided (Fig. 2, Supplementary Figs. S2 and S3). Since the image analysis method for immunostaining differed between biopsy specimens and surgical specimens, they were analyzed separately.

### Evaluations of TLS density

To assess TLS density, we identified dense lymphatic aggregates in the whole tumor area and counted their numbers in H&E-stained sections. For quantitative evaluation, we scanned whole slides and used the same definition for the tumor area margin as described above, within which we found TLSs to be exclusively formed in our preliminary experiment (Fig. 1E). The area of each peritumoral region was measured using HALO software. We defined TLS density as the number of TLSs per millimeter in the tumor area margin and used the median as the cutoff value to categorize them.

### Statistical analysis

Continuous variables are expressed as median and range and were analyzed using Mann–Whitney U test. Survival time was evaluated using Kaplan–Meier method, with comparisons made using log-rank test. The Cox proportional hazard model was used for calculating hazard ratios (HR) and for univariate/multivariate analysis. Progression-free survival (PFS) was defined as the interval from the first nivolumab administration to disease progression or death. Overall survival (OS) was defined as the interval from the first nivolumab administration to death from any cause. Prognostic variables showing significant association in univariate analysis were included in multivariate analysis. All P values were two-sided, with *P* < 0.05 indicating statistical significance. Statistical analyses were performed using JMP Pro software (version 16.2.0; SAS Institute Inc., Cary, NC, USA).

## Results

### Patient backgrounds and outcomes with nivolumab treatment

Baseline characteristics are listed in Table S1. Of the 250 patients, 193 (77.2%) were male, and the median age at ICI initiation was 70 (range 32–89). All enrolled patients had ESCC, and almost half (48.0%) had an Eastern Cooperative Oncology Group PS0. Of the overall cohort, 131 (52.4%) patients had unresectable advanced ESCC, and 119 (47.6%) had recurrent disease. All patients had received previous systemic anticancer therapy, with 133 (53.2%) undergoing surgery and 105 (42.7%) undergoing radiotherapy. The evaluated samples comprised endoscopic biopsies (n = 121, 48.4%) and surgical specimens (n = 129, 51.6%) before ICI administration. The status distributions were CR in 5 (2.0%), PR in 31 (12.4%), SD in 80 (32.0%) patients, and progressive disease (PD) in 134 (53.6%) patients, for an objective response rate of 14.4% (36/250) and a disease control rate of 46.4% (116/250).

### Relationship of CD3^+^, CD8^+^, and Foxp3^+^lymphocyte counts and nivolumab response in surgical specimens

**Fig. 3:**
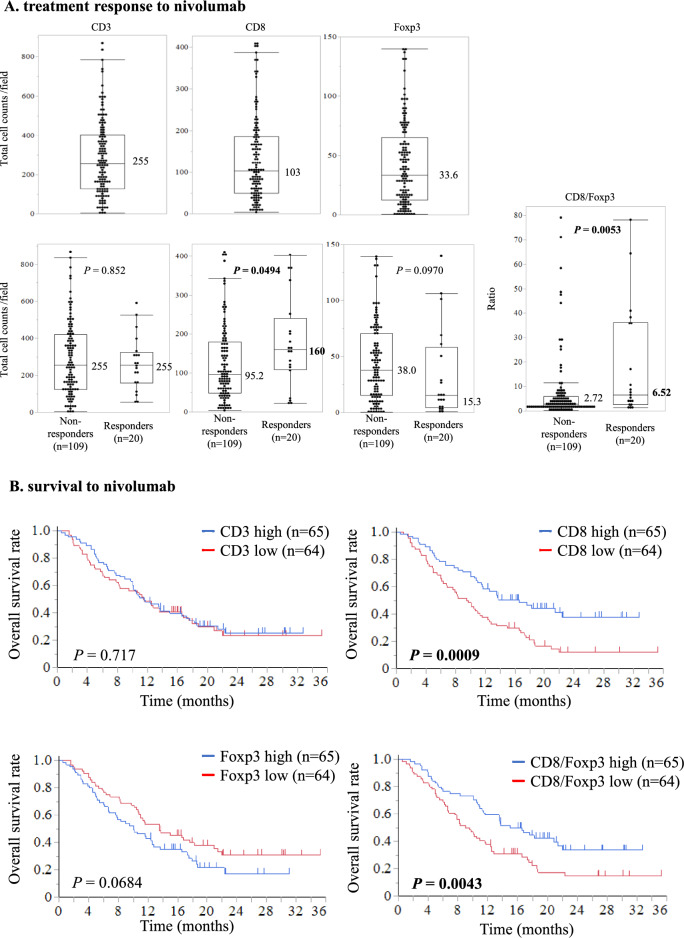
Relationship between counts of CD3^+^, CD8 ^+^, and Foxp3^+^ lymphocytes and treatment response and survival with nivolumab in surgical specimens. **A** top: CD3^+^, CD8^+^, and Foxp3^+^ lymphocyte counts. **A** Middle: lymphocyte counts according to treatment response to nivolumab. **A** Bottom: CD8/Foxp3 ratio according to treatment response to nivolumab. Values in boxplots indicate medians. **B** Kaplan–Meier survival curves for Overall survival according to CD3^+^, CD8^+^, and Foxp3^+^ lymphocyte counts and CD8/Foxp3 ratio

Among 129 patients with surgical specimens, 109 (84.5%) showed a non-response (SD/PD) and 20 (15.5%) showed a response (CR/PR) to treatment. Surgical specimens were analyzed based on CD3^+^, CD8^+^, and Foxp3^+^lymphocyte counts in the defined tumor area. The median values for cells per field were 255 for CD3^+^, 103 for CD8^+^, and 33.6 for Foxp3^+^ (Fig. 3A). The number of CD8^+^ lymphocytes predicted response vs. non-response to nivolumab (respectively 160 vs. 95.2 cells/field, *P* = 0.0494), but the CD3^+^ lymphocyte count did not correlate with response vs. non-response to nivolumab (respectively 255 vs. 255 cells/field, *P* = 0.852). The number of Foxp3^+^ lymphocytes per field showed a trend to an association with nivolumab response (15.3 with response vs. 38.0 with non-response, *P* = 0.0970). Remarkably, the CD8/Foxp3 ratio significantly correlated with response vs. non-response to nivolumab (respectively 6.52 vs. 2.72, *P* = 0.0053).

### Patient survival according to CD3^+^, CD8^+^, and Foxp3^+^ lymphocyte counts in surgical specimens

Regarding survival analysis of the cohort with surgical specimens, the median follow-up for the censored patients was 2.5 months for PFS and 11.6 months for OS. Compared with a low-CD8^+^ count, a high CD8^+^ count was significantly associated with better median PFS (2.9 vs. 1.7 months, *P* = 0.0181) and OS (16.5 vs. 9.5 months, *P* = 0.0009). Similarly, compared with a low CD8/Foxp3 ratio, a high CD8/Foxp3 ratio was associated with better median PFS (3.0 vs. 1.9 months, *P* = 0.0090) and OS (13.8 vs. 9.0 months, *P* = 0.0043; Fig. 3B and Supplementary Fig. S4A). Meanwhile, we found no significant correlation of OS with CD3^+^ (*P* = 0.717) or Foxp3^+^ (*P* = 0.0684) counts.

### Patient survival and nivolumab response by TLS density in surgical specimens

**Fig. 4:**
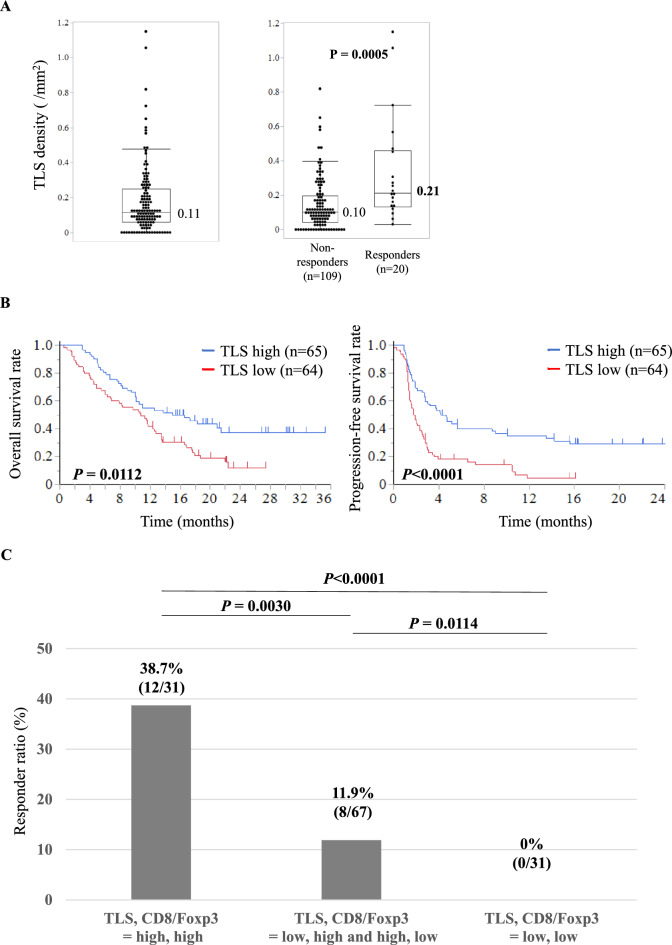
Relation between TLS expression and treatment response and prognosis with nivolumab. **A** TLS density (left) and TLS density according to treatment response to nivolumab (right); values in boxplots are medians. **B** Kaplan–Meier survival curves for progression-free and overall survival according to TLS density. **C** The response rates with nivolumab in three groups representing various combinations of high versus low TLS density and CD8/Foxp3 ratio

Among 129 patients with surgical specimens, the median TLS density was 0.11/mm^2^ (Fig. 4A). TLS density predicted response vs. non-response to nivolumab (respectively 0.21 vs. 0.10/mm^2^, *P* = 0.0005). In survival analysis, a high vs. low TLS density was associated with better median OS (13.2 vs. 10.7 months, *P* = 0.0112) and PFS (4.1 vs. 1.7 months, *P* < 0.0001) (Fig. 4B). When patients were categorized into three groups by TLS density and CD8/Foxp3 ratio, response rates were 38.7% in the high/high group, 11.9% in the low/high and high/low groups, and 0% in the low/low group (Fig. 4C).

### Nivolumab response and survival by CD3^+^, CD8^+^, Foxp3^+^, and CCR8^+^ lymphocyte counts in endoscopic biopsies

**Fig. 5:**
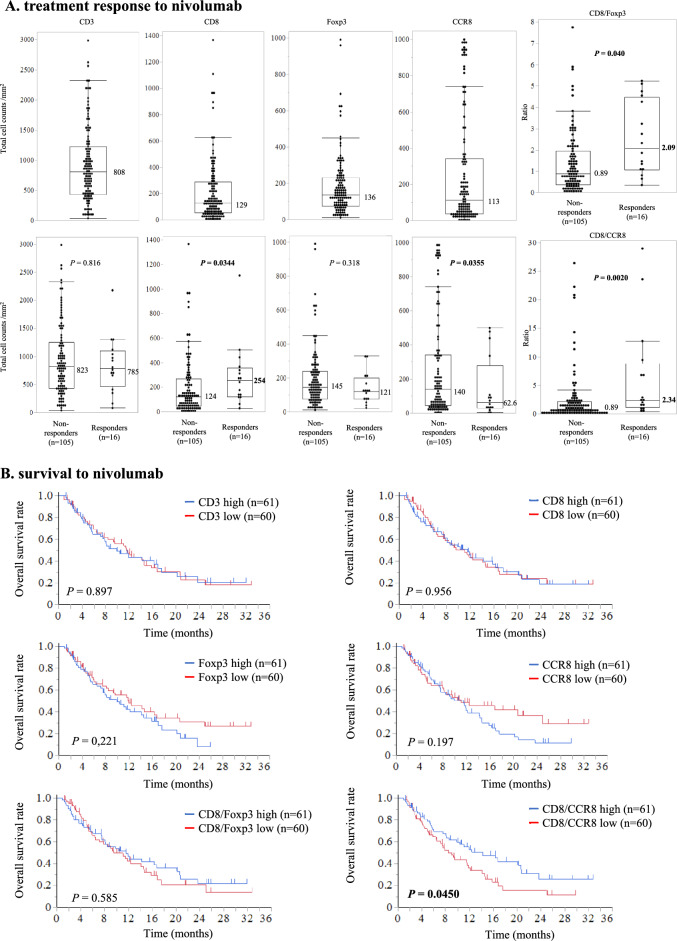
Relation between CD3^+^, CD8^+^, Foxp3^+^, and CCR8^+^ lymphocyte counts in endoscopic biopsies and treatment response and survival with nivolumab. **A** Top: CD3^+^, CD8^+^, Foxp3^+^, and CCR8^+^ lymphocyte counts. **A** Middle: lymphocyte counts according to treatment response to nivolumab. **A** Bottom: CD8/Foxp3 and CD8/CCR8 ratios according to treatment response to nivolumab. Values in boxplots are medians. **B** Kaplan–Meier survival curves for Overall survival according to the lymphocyte counts and CD8/Foxp3 and CD8/CCR8 ratios

Among 121 patients with an endoscopic biopsy available for evaluation, 105 (86.8%) had a non-response and 16 (23.2%) experienced a response. Median CD3^+^, CD8^+^, Foxp3^+^, and CCR8^+^ lymphocyte counts were respectively 808, 129, 136, and 113 cells/mm^2^ (Fig. 5A). CD8^+^ and CCR8^+^ lymphocyte counts significantly correlated with response to nivolumab (response vs. non-response: 254 vs. 124 CD8^+^ cells/mm^2^, *P* = 0.0344; 62.6 vs. 140 CCR8^+^ cells/mm^2^, *P* = 0.0355). On the other hand, the number of CD3^+^ and Foxp3^+^ lymphocytes did not correlate with nivolumab response (response vs. non-response: 785 vs. 823 CD3^+^ cells/field, *P* = 0.816; 121 vs. 145 Foxp3^+^ cells/field, *P* = 0.318). Intriguingly, the CD8/Foxp3 and CD8/CCR8 ratios significantly correlated with response vs. non-response to nivolumab (CD8/Foxp3: 2.09 vs. 0.89, *P* = 0.040; CD8/CCR8: 2.34 vs. 0.89, *P* = 0.0020).

Median follow-up for censored patients with endoscopic biopsy samples was 2.3 months for PFS and 11.1 months for OS. Compared with a low CD8/CCR8 ratio, a high ratio was associated with better median PFS (2.6 vs. 1.3 months, *P* = 0.0092) and OS (12.9 vs. 8.4 months, *P* = 0.0450; Fig. 5B and Supplementary Fig. S4B).

### Univariate and multivariate analyses of patient survival and nivolumab response according to surgical specimen and endoscopic biopsy data

Univariate analysis of data from surgical specimens revealed that OS was significantly associated with age, sex, performance status, history of smoking, previous surgery, previous radiotherapy, number of previous chemotherapy regimens, number of organs affected by metastasis, CD3^+^, CD8/Foxp3 ratio, and TLS (Table 1). Multivariate analysis further identified CD8/Foxp3 ratio (HR = 1.83, 95% confidence interval [CI] 1.20–2.81, *P* = 0.0050) and TLS density (HR = 1.67, 95% CI 1.09–2.57, *P* = 0.0171) as independent prognostic parameters. Similarly, univariate analysis of biopsy data showed a significant association of PFS with age, sex, performance status, history of smoking, previous surgery, previous radiotherapy, number of previous chemotherapy regimens, number of organs affected by metastasis, CD3^+^, CD8/Foxp3 ratio, and CD8/CCR8 ratio. In the multivariate analysis, CD8/CCR8 ratio (HR = 1.66, 95% CI 1.04–2.65, *P* = 0.0313) was further identified as an independent prognostic parameter. Results were similar between univariate and multivariate analyses for PFS and nivolumab response (Tables S2, S3, S4 and S5).

**Table 1:** Univariate and multivariate analyses for OS

		Surgical specimens						Endoscopic biopsies			
Variables	Category	Univariate analysis		Multivariate analysis		Variables	Category	Univariate analysis		Multivariate analysis	
		HR (95% CI)	*P*	HR (95% CI)	*P*			HR (95% CI)	*P*	HR (95% CI)	*P*
Age (y)	≤70	1.17 (0.78–1.77)	0.431			Age (y)	≤70	0.89 (0.56–1.41)	0.621		
Sex	Female	0.41 (0.87–2.27)	0.162			Sex	Female	1.22 (0.73–2.02)	0.441		
Performance status	1–3	1.98 (1.31–3.01)	**0.0012**	1.68 (1.09–2.58)	**0.0173**	Performance status	1–3	1.65 (1.04–2.62)	**0.0334**	1.71 (1.07–2.72)	**0.0224**
History of smoking	Yes	0.91 (0.57–1.43)	0.695			History of smoking	Yes	0.71 (0.43–1.19)	0.201		
Previous surgery	No	1.17 (0.47–2.88)	0.732			Previous surgery	No	1.12 (0.51–2.45)	0.780		
Previous radiotherapy	Yes	0.89 (0.58–1.39)	0.630			Previous radiotherapy	Yes	1.03 (0.64–1.64)	0.886		
Number of previous chemotherapy regimens	3-	1.20 (0.69–2.10)	0.505			Number of previous chemotherapy regimens	3-	1.25 (0.72–2.18)	0.422		
Number of organs with metastases	3-	1.33 (0.79–2.23)	0.272			Number of organs with metastases	3-	1.48 (0.84–2.63)	0.173		
CD3	Low	1.07 (0.71–1.62)	0.716			CD3	Low	0.99 (0.63–1.57)	0.982		
CD8/Foxp3	Low	1.82 (1.20–2.78)	**0.0049**	1.83 (1.20–2.81)	**0.0050**	CD8/Foxp3	Low	1.13 (0.71–1.79)	0.585		
TLS	Low	1.69 (1.12–2.56)	**0.0122**	1.67 (1.09–2.57)	**0.0171**	CD8/CCR8	Low	1.59 (1.00–2.53)	**0.0469**	1.66 (1.04–2.65)	**0.0313**

Bold text indicates a *P*-value of less than 0.05

*CI* confidence interval, *HR* hazard ratio, *PFS* progression-free survival

## Discussion

In this study, we quantitatively assessed the tumor immune microenvironment using IHC on endoscopic and surgical specimens of ESCCs taken before nivolumab administration. In surgical specimens, CD8^+^ cell count, CD8/Foxp3 ratio, and TLS density were useful in predicting therapeutic efficacy and patient prognosis, with the CD8/Foxp3 ratio and TLS density identified as independent prognostic factors. In biopsy specimens, CD8^+^ and CCR8^+^ cells and CD8/Foxp3 and CD8/CCR8 ratios predicted therapeutic efficacy, with the CD8/CCR8 ratio emerging as an important prognostic parameter. We previously found that CAR (C-reactive protein: albumin ratio) and PS (performance status) before nivolumab treatment are useful in predicting long-term survival in patients with recurrent/unresectable advanced ESCC undergoing second-line or later nivolumab treatment [25]. To our knowledge, this multicenter study, involving 250 clinical samples from 15 institutions of the Clinical Study Group of Osaka University, represents the most extensive biomarker investigation of nivolumab for unresectable or recurrent ESCC.

Moreover, this study is the first to objectively assess IHC results using the automated cell count system HALO. Recently, digital automated image analysis in IHC has become essential for quantitative evaluations [26]. The AI-powered HALO facilitates accurate analysis of histopathological changes that are difficult to assess manually [27]. This automated approach enhances the objectivity, precision, and persuasiveness of pathological evaluations. In this study, we used HALO for objective evaluation of IHC/H&E staining. We previously reported a significant correlation between prognosis and the immunoscore, determined by counting TILs in the tumor core and invasive margin, in preoperatively untreated esophageal cancer [17]. In this study, we focused on TILs at the tumor area margin of 1000 μm in surgical specimens.

CD8^+^ cytotoxic T cell count has been identified as a biomarker for ICI therapy in melanoma, breast cancer, and lung cancer [28–34]. Additionally, the localization of CD8 expression, as assessed by IHC, has been recognized as a biomarker for ICI therapy in breast cancer [34]. In gastric cancer patients treated with ICIs, hyperprogressive disease cases exhibit high infiltration of effector regulatory T cells (Tregs) at the tumor site [35]. This study focused on CCR8, a marker of effector Tregs, since CCR8^+^ Tregs are specifically found within tumors and high CCR8 expression is linked to poor prognosis [36]. Consistent with reports in other cancers, this study identified CD8 expression in the local tumor environment of esophageal cancer as a biomarker for ICI therapy effects. The study also showed that CCR8 expression in biopsy specimens before ICI therapy correlated with treatment efficacy, and low CCR8/low Foxp3 was associated with the best prognosis among the four classifications based on high or low CCR8 and Foxp3 expression (Supplementary Figure S5). These results suggest a role for effector Tregs in the ICI therapy response in ESCC. In our previous report, CCR8^+^ Tregs made up ~ 40% of the total Treg population within the tumor [36]. However, in this study, the median number (113 cells/mm^2^) of CCR8^+^ lymphocytes were relatively high compared to the median number (136 cells/mm^2^) of Foxp3^+^ lymphocytes in biopsy specimens. This difference may be due to findings that CCR8 is expressed not only in Tregs but also in other immune cells, including Th2 cells [37].

In evaluating the immune microenvironment, it is crucial to assess the “positive and negative” balance of antitumor immunity, beyond single parameters. In various cancers, including renal cell carcinoma, melanoma, and breast, gastric, ovarian, and cervical cancers, a decreased CD8^+^ cell to Treg ratio correlates with poor prognosis [38–40]. Additionally, in gastric and lung cancers, the CD8 to Foxp3 relationship is associated with ICI therapy effectiveness [41, 42]. Although, in the current study, Foxp3 alone did not predict efficacy or prognosis with ICI treatment, the assessment of both CD8 and Foxp3 significantly correlated with ICI treatment efficacy. In vitro, Tregs reportedly suppress nivolumab-induced interferon-γ release by effector T cells [14]. Given our earlier report of a correlation between poor prognosis and a lower CD8^+^ cell to Treg ratio in pre-treated esophageal cancer patients, Tregs in esophageal cancer, like in other solid tumors, may significantly impact immunotherapy efficacy by inhibiting the cytotoxic activity of CD8 associated with the tumor [43].

We previously showed that high peritumoral TLS density in the primary tumor predicted response to later anti–PD-1 antibody treatment and subsequent survival, highlighting the clinical benefit of TLS evaluation in initial specimens from patients with recurrent esophageal cancer [23]. In this larger multicenter study, TLS density proved to be an independent predictor of prognosis and therapeutic effects of ICIs, confirming earlier results. A possible mechanism could be that high peritumoral TLS density is associated with increased tumor-associated memory B cells, plasma cells, or PD-1(+) immune infiltrates, enhancing ICI therapy response [44, 45]. Our previous study evaluated TLS maturity using immunostaining with H&E staining, but this work focused mainly on TLS expression assessed by H&E staining, which is more applicable to clinical settings. Indeed, TLS density in resected specimens may guide postoperative adjuvant nivolumab therapy in esophageal cancer. As TLS assessment is not feasible for biopsy specimens, alternative biomarkers like CCR8 may be necessary for biopsy-based cases. Although previous reports noted a correlation between CD8^+^ infiltrates within TLSs and prognosis or ICI treatment efficacy, this study found that combining TLS with the CD8/Foxp3 ratio was a more sensitive predictor of ICI treatment effectiveness [46, 47]. Thus, combining future therapies like CAR-T and TLS induction may further improve ICI efficacy.

This study had several limitations. First, it used a retrospective design, and the treatment modalities and patient follow-up schedules were not strictly standardized across facilities and physicians. However, data for consecutive ESCC patients treated with nivolumab were obtained from each institution, minimizing selection bias. Second, the study showed differences in CD3, CD8, and Foxp3 expression rates between biopsy and surgical specimens and identified some variation in ICI biomarker results. These variations are partly due to potential heterogeneity in immunostaining and different IHC evaluation methods between biopsy and surgical specimens. Additionally, defining the “tumor margin” in surgical specimens is challenging, as residual tumor cells may scatter due to preoperative chemotherapy. Changes in the tumor microenvironment during treatment, including PD-L1 expression and TILs, could have significantly affected the results [48, 49]. Nevertheless, CD8 consistently proved to be an important biomarker of ICI efficacy in both biopsy and surgical specimens, confirming the utility of cytotoxic T cell evaluation in predicting ICI efficacy. Third, this study did not specifically address the clinical significance of PD-L1 expression but evaluated the association of the combined positive score/tumor proportion score with efficacy and survival outcomes with nivolumab. The results indicated that patients responding to therapy had a high tumor proportion score (≥ 1%) and combined positive score (≥ 10%), and patients with a high combined positive score (≥ 10%) had significantly more favorable PFS (data not shown). In the future, combining host factors with PD-L1 expression may allow for more precise predictions of treatment effects.

In conclusion, this multicenter biomarker study utilized the automated analysis software HALO to objectively assess TILs in a large cohort of patients with unresectable or recurrent ESCC. The results indicate that CD8^+^ and CCR8^+^ cell counts, CD8/Foxp3 and CD8/CCR8 ratios, and TLS density could serve as predictive markers for ICI therapy in ESCC patients. Although these findings should be validated with independent cohort samples, combining these factors to develop a scoring system may help establish personalized treatment strategies for ESCC patients.

## Supplementary Information

Below is the link to the electronic supplementary material.

Supplementary file1 (PPTX 1805 KB)

Supplementary file2 (PPTX 1648 KB)

Supplementary file3 (PPTX 2312 KB)

Supplementary file4 (PPTX 52 KB)

Supplementary file5 (PPTX 60 KB)

Supplementary file6 (PPTX 54 KB)

Supplementary file7 (DOCX 200 KB)

Supplementary file8 (DOCX 201 KB)

Supplementary file9 (DOCX 201 KB)

Supplementary file10 (DOCX 201 KB)

Supplementary file11 (DOCX 201 KB)

## Data Availability

The data generated in this study are available upon request from the corresponding author.
