# Supplementary material for: Exploring predictive biomarkers of efficacy and survival with nivolumab treatment for unresectable/recurrent esophageal squamous cell carcinoma
Source: Esophagus. 2025 Apr 24;22(3):360–72. doi: 10.1007/s10388-025-01120-z (PMC12167336; doi:10.1007/s10388-025-01120-z)
Supplement: Supplementary file 1 — Supplementary file1 (PPTX 1805 KB) [file 10388_2025_1120_MOESM1_ESM.pptx]

## Slide 1
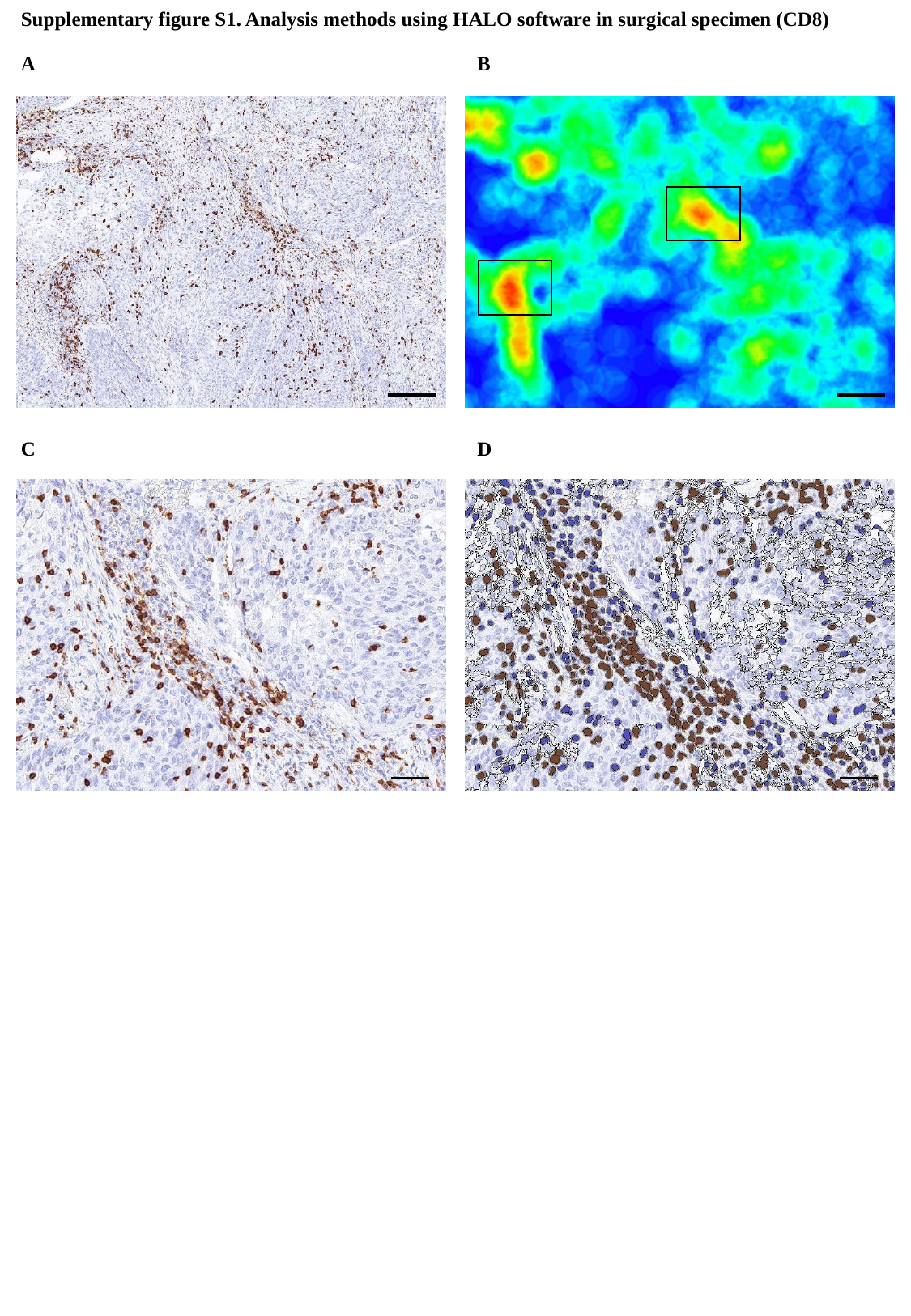

Supplementary figure S1. Analysis methods using HALO software in surgical specimen (CD8)
A
B
C
D
