# Supplementary material for: Exploring predictive biomarkers of efficacy and survival with nivolumab treatment for unresectable/recurrent esophageal squamous cell carcinoma
Source: Esophagus. 2025 Apr 24;22(3):360–72. doi: 10.1007/s10388-025-01120-z (PMC12167336; doi:10.1007/s10388-025-01120-z)
Supplement: Supplementary file 2 — Supplementary file2 (PPTX 1648 KB) [file 10388_2025_1120_MOESM2_ESM.pptx]

## Slide 1
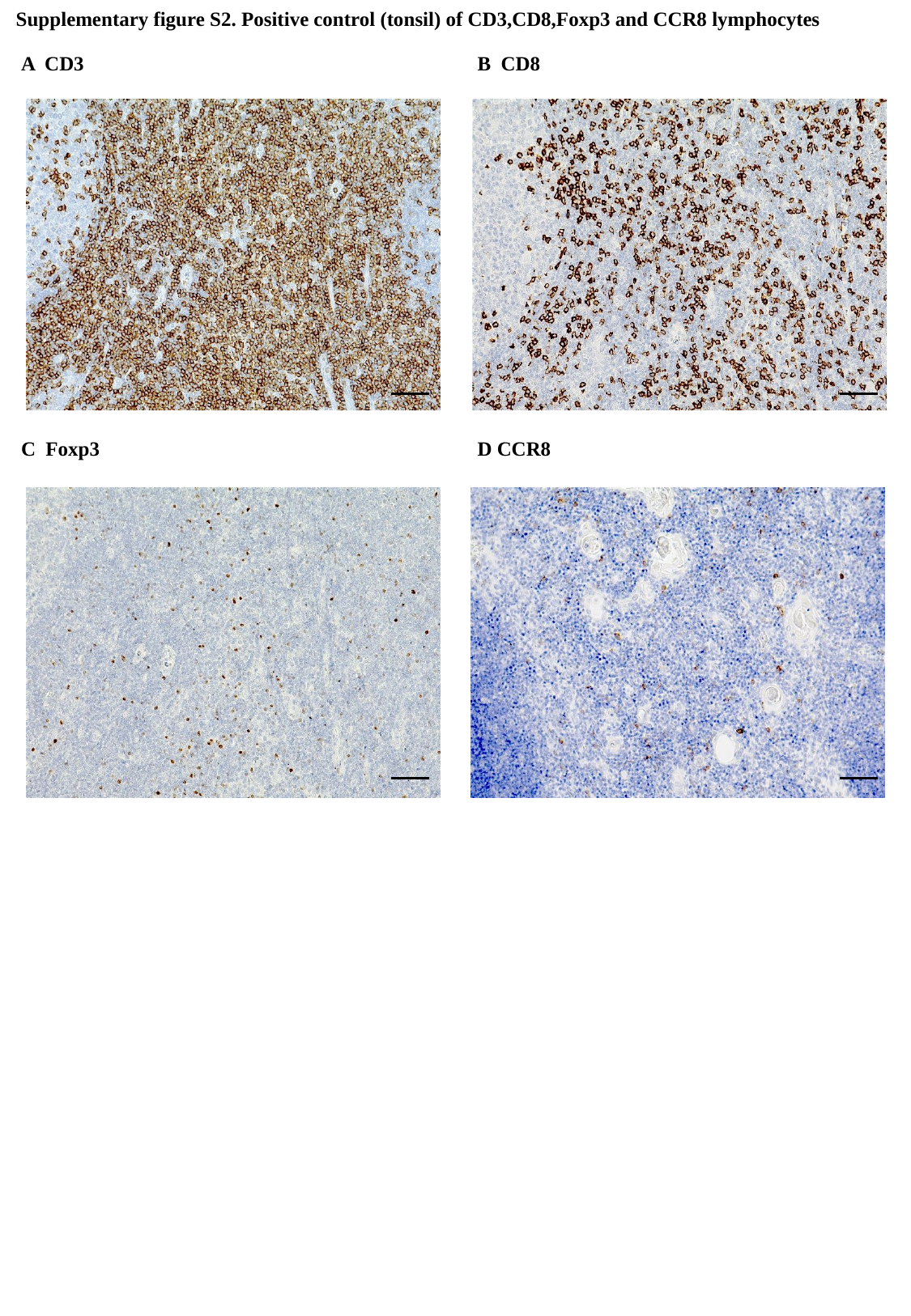

Supplementary figure S2. Positive control (tonsil) of CD3,CD8,Foxp3 and CCR8 lymphocytes
A CD3
B CD8
C Foxp3
D CCR8
