# Supplementary material for: Exploring predictive biomarkers of efficacy and survival with nivolumab treatment for unresectable/recurrent esophageal squamous cell carcinoma
Source: Esophagus. 2025 Apr 24;22(3):360–72. doi: 10.1007/s10388-025-01120-z (PMC12167336; doi:10.1007/s10388-025-01120-z)
Supplement: Supplementary file 3 — Supplementary file3 (PPTX 2312 KB) [file 10388_2025_1120_MOESM3_ESM.pptx]

## Slide 1
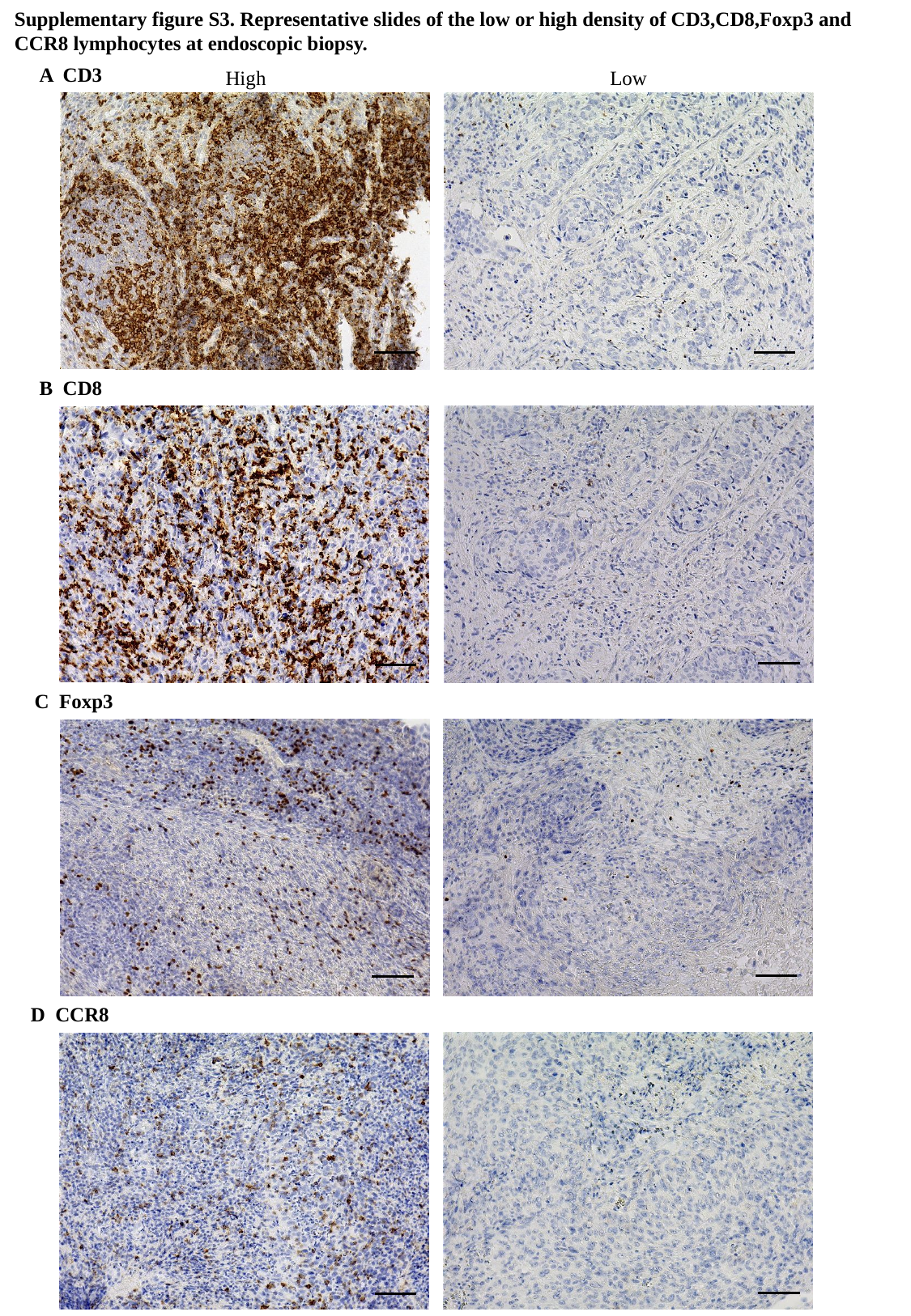

Supplementary figure S3. Representative slides of the low or high density of CD3,CD8,Foxp3 and CCR8 lymphocytes at endoscopic biopsy.
A CD3
High
Low
B CD8
C Foxp3
D CCR8
