# Supplementary material for: Exploring predictive biomarkers of efficacy and survival with nivolumab treatment for unresectable/recurrent esophageal squamous cell carcinoma
Source: Esophagus. 2025 Apr 24;22(3):360–72. doi: 10.1007/s10388-025-01120-z (PMC12167336; doi:10.1007/s10388-025-01120-z)
Supplement: Supplementary file 4 — Supplementary file4 (PPTX 52 KB) [file 10388_2025_1120_MOESM4_ESM.pptx]

## Slide 1
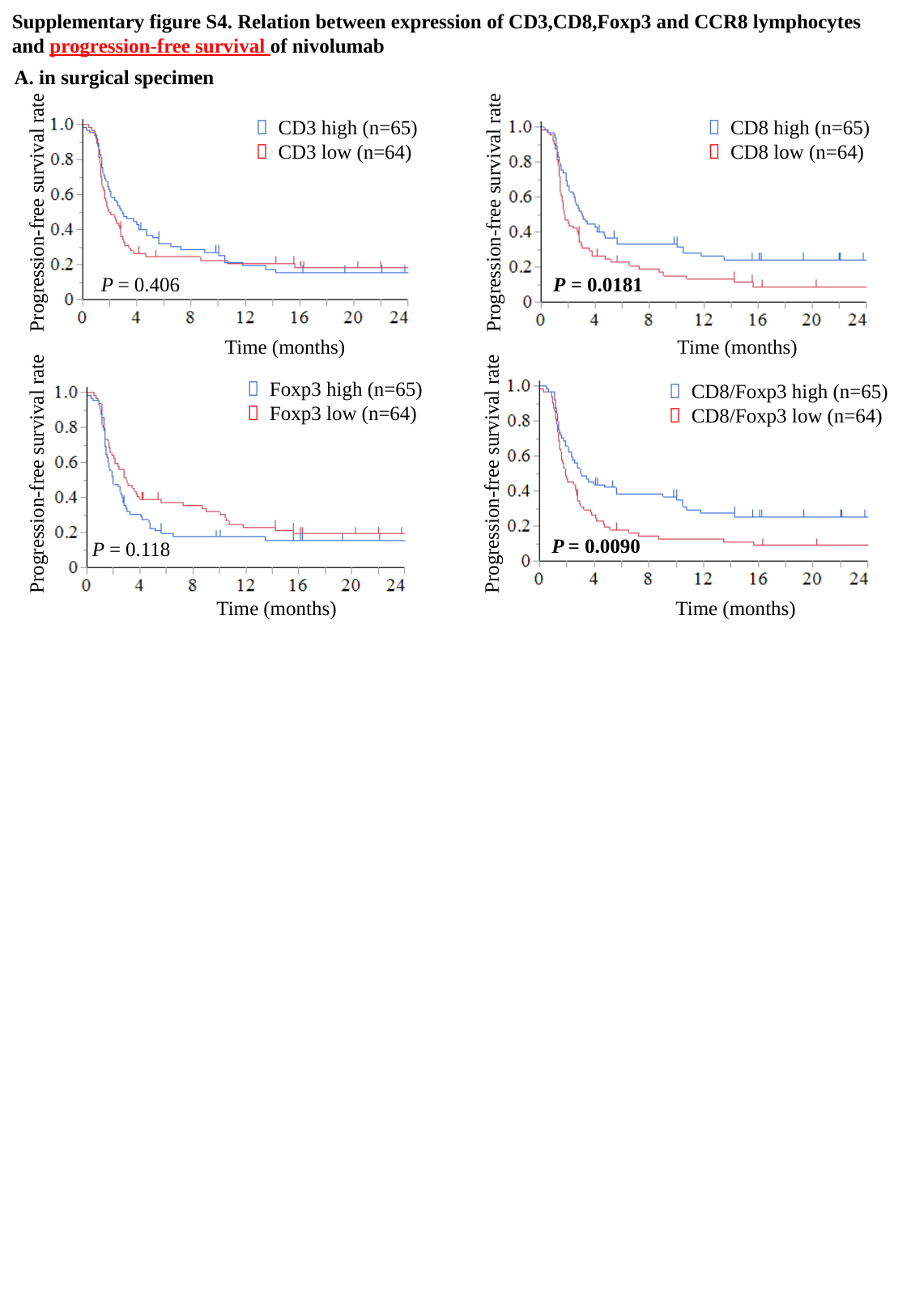

Supplementary figure S4. Relation between expression of CD3,CD8,Foxp3 and CCR8 lymphocytes and progression-free survival of nivolumab
A. in surgical specimen
ー CD3 high (n=65)
ー CD3 low (n=64)
ー CD8 high (n=65)
ー CD8 low (n=64)
Progression-free survival rate
Progression-free survival rate
P = 0.406
P = 0.0181
Time (months)
Time (months)
ー Foxp3 high (n=65)
ー Foxp3 low (n=64)
ー CD8/Foxp3 high (n=65)
ー CD8/Foxp3 low (n=64)
Progression-free survival rate
Progression-free survival rate
P = 0.0090
P = 0.118
Time (months)
Time (months)
