# Supplementary material for: Exploring predictive biomarkers of efficacy and survival with nivolumab treatment for unresectable/recurrent esophageal squamous cell carcinoma
Source: Esophagus. 2025 Apr 24;22(3):360–72. doi: 10.1007/s10388-025-01120-z (PMC12167336; doi:10.1007/s10388-025-01120-z)
Supplement: Supplementary file 5 — Supplementary file5 (PPTX 60 KB) [file 10388_2025_1120_MOESM5_ESM.pptx]

## Slide 1
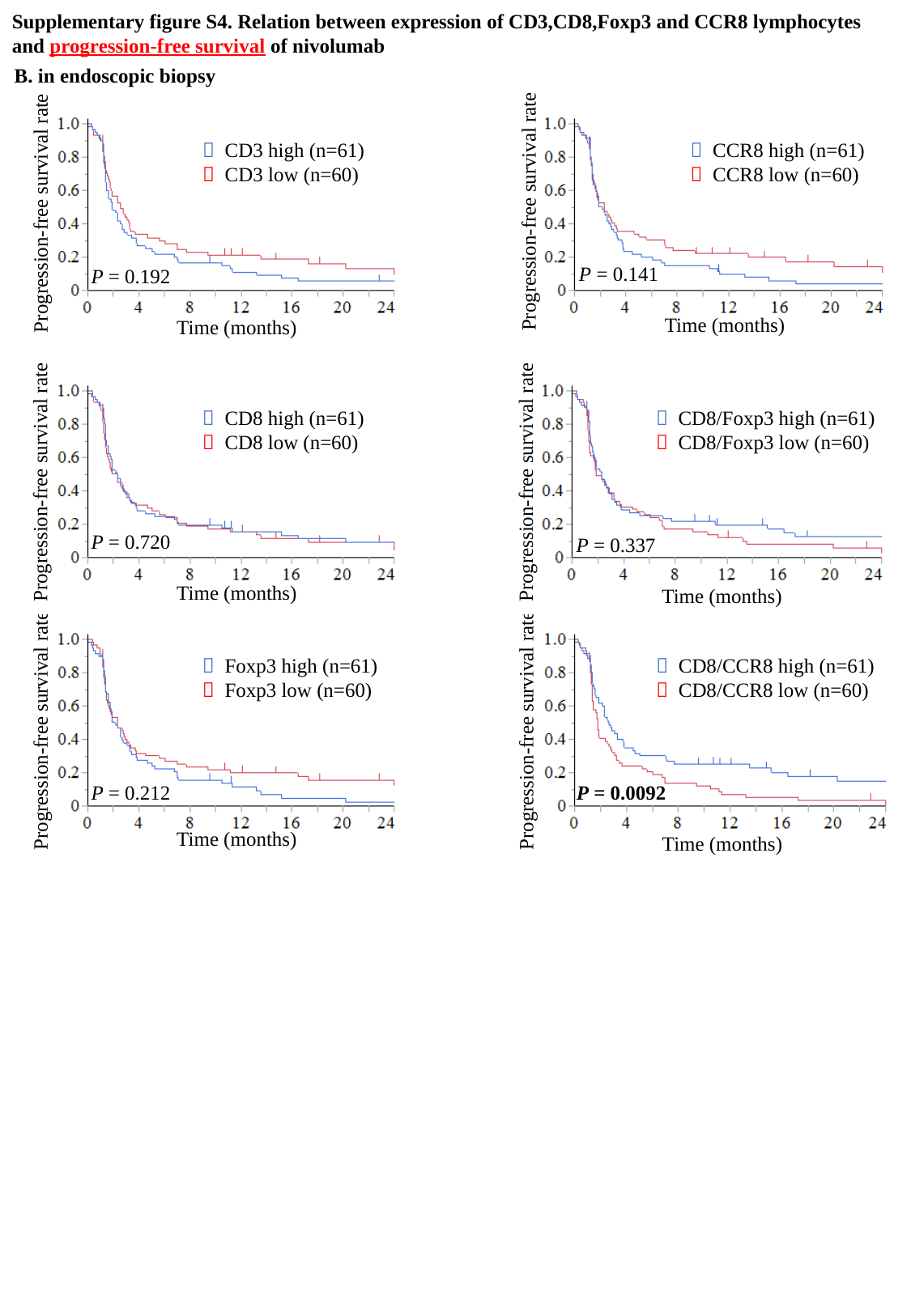

Supplementary figure S4. Relation between expression of CD3,CD8,Foxp3 and CCR8 lymphocytes and progression-free survival of nivolumab
B. in endoscopic biopsy
ー CD3 high (n=61)
ー CD3 low (n=60)
ー CCR8 high (n=61)
ー CCR8 low (n=60)
Progression-free survival rate
Progression-free survival rate
P = 0.141
P = 0.192
Time (months)
Time (months)
ー CD8 high (n=61)
ー CD8 low (n=60)
ー CD8/Foxp3 high (n=61)
ー CD8/Foxp3 low (n=60)
Progression-free survival rate
Progression-free survival rate
P = 0.720
P = 0.337
Time (months)
Time (months)
ー Foxp3 high (n=61)
ー Foxp3 low (n=60)
ー CD8/CCR8 high (n=61)
ー CD8/CCR8 low (n=60)
Progression-free survival rate
Progression-free survival rate
P = 0.212
P = 0.0092
Time (months)
Time (months)
