# Supplementary material for: Exploring predictive biomarkers of efficacy and survival with nivolumab treatment for unresectable/recurrent esophageal squamous cell carcinoma
Source: Esophagus. 2025 Apr 24;22(3):360–72. doi: 10.1007/s10388-025-01120-z (PMC12167336; doi:10.1007/s10388-025-01120-z)
Supplement: Supplementary file 6 — Supplementary file6 (PPTX 54 KB) [file 10388_2025_1120_MOESM6_ESM.pptx]

## Slide 1
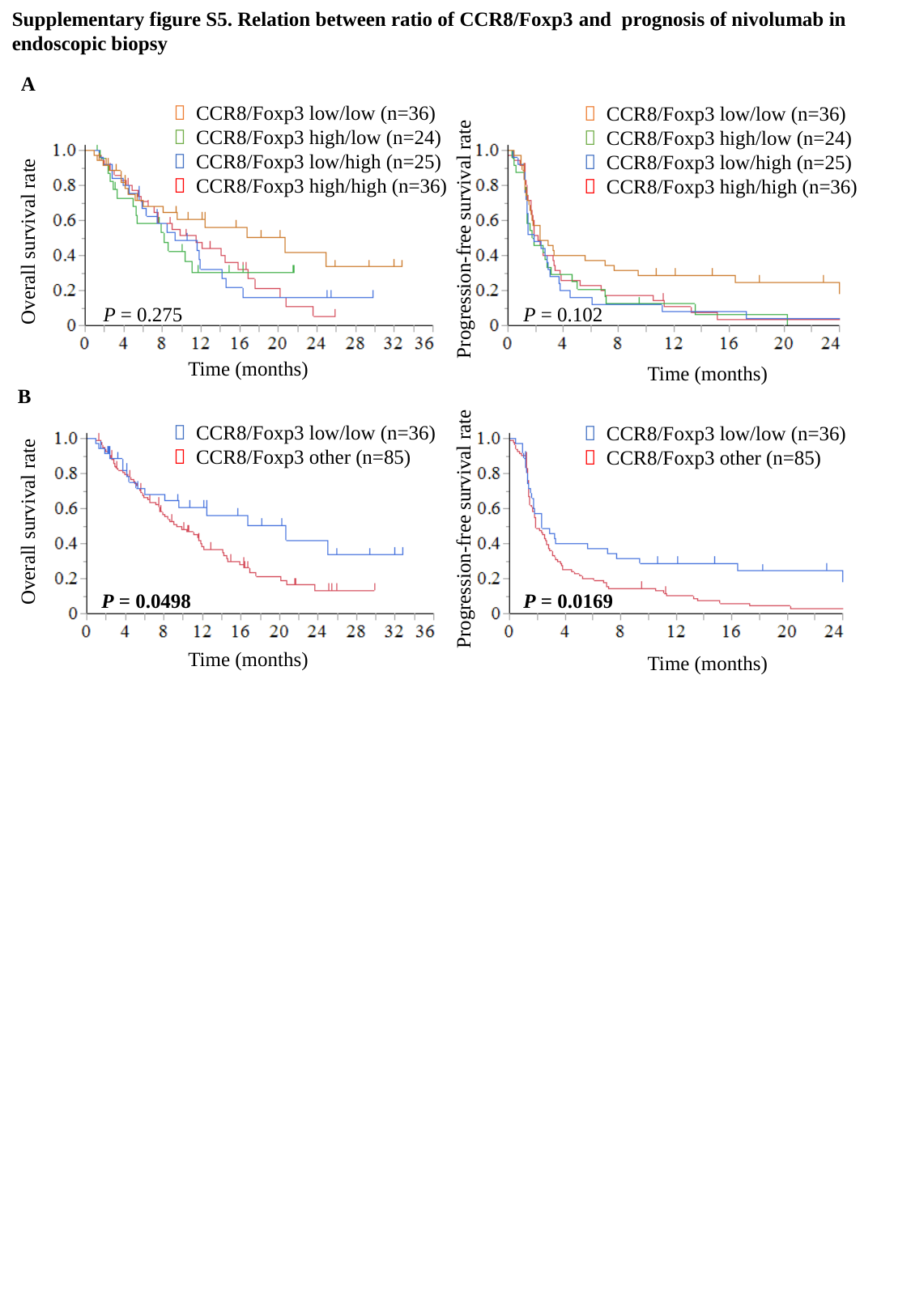

Supplementary figure S5. Relation between ratio of CCR8/Foxp3 and prognosis of nivolumab in endoscopic biopsy
A
ー CCR8/Foxp3 low/low (n=36)
ー CCR8/Foxp3 high/low (n=24)
ー CCR8/Foxp3 low/high (n=25)
ー CCR8/Foxp3 high/high (n=36)
ー CCR8/Foxp3 low/low (n=36)
ー CCR8/Foxp3 high/low (n=24)
ー CCR8/Foxp3 low/high (n=25)
ー CCR8/Foxp3 high/high (n=36)
Progression-free survival rate
Overall survival rate
P = 0.275
P = 0.102
Time (months)
Time (months)
B
ー CCR8/Foxp3 low/low (n=36)
ー CCR8/Foxp3 other (n=85)
ー CCR8/Foxp3 low/low (n=36)
ー CCR8/Foxp3 other (n=85)
Overall survival rate
Progression-free survival rate
P = 0.0498
P = 0.0169
Time (months)
Time (months)
