# Supplementary material for: Exploring predictive biomarkers of efficacy and survival with nivolumab treatment for unresectable/recurrent esophageal squamous cell carcinoma
Source: Esophagus. 2025 Apr 24;22(3):360–72. doi: 10.1007/s10388-025-01120-z (PMC12167336; doi:10.1007/s10388-025-01120-z)
Supplement: Supplementary file 7 — Supplementary file7 (DOCX 200 KB) [file 10388_2025_1120_MOESM7_ESM.docx]

Supplementary table S1. Patient characteristics

| Characteristics, n (%) | N = 250 |
| --- | --- |
| Age  　Median (range), years | 70（32–89) |
| Sex  　Male/female | 193 (77.2)/57 (22.8) |
| Performance status  　0/1/2/3 | 120 (48.0)/112 (44.8)/16 (6.4)/2 (0.8) |
| History of smoking  　Yes/no | 184 (73.6)/66 (26.4) |
| Unresectable/recurrent | 131 (52.4)/119 (47.6) |
| Number of previous chemotherapy regimens  　0/1/2/3- | 7 (2.8)/142 (56.8)/64 (25.6)/37 (14.8) |
| Number of organs with metastases  　0/1/2/3- | 12 (4.8)/117 (46.8)/77 (30.8)/44 (17.6) |
| Previous surgery  　Yes/no | 133 (53.2)/117 (46.8) |
| Previous radiotherapy  　Yes/no | 107 (42.8)/143 (57.2) |
| Specimen  　Surgical/biopsy | 129 (51.6)/121 (48.4) |
| Response rate  　Complete response  　Partial response  　Stable disease  　Progressive disease  Objective response  Disease control | 5 (2.0)  31 (12.4)  80 (32.0)  134 (53.6)  36 (14.4)  116 (46.4) |
