# Supplementary material for: Exploring predictive biomarkers of efficacy and survival with nivolumab treatment for unresectable/recurrent esophageal squamous cell carcinoma
Source: Esophagus. 2025 Apr 24;22(3):360–72. doi: 10.1007/s10388-025-01120-z (PMC12167336; doi:10.1007/s10388-025-01120-z)
Supplement: Supplementary file 8 — Supplementary file8 (DOCX 201 KB) [file 10388_2025_1120_MOESM8_ESM.docx]

Supplementary table S2. Univariate and multivariate analyses for non-response surgical specimens (n = 129)

| Variables | Category | Univariate analysis | | | Multivariate　analysis |
| --- | --- | --- | --- | --- | --- |
|  |  | HR (95% CI) | *P* | HR (95% CI) | *P* |
| Age (years) | ≤70 | 1.49  (0.57–3.88) | 0.413 |  |  |
| Sex | Female | 1.34  (0.44–4.11) | 0.599 |  |  |
| Performance status | 1–3 | 2.26  (0.84–6.10) | 0.106 | 1.65  (0.56–4.85) | 0.362 |
| History of smoking | Yes | 1.13  (0.37–3.41) | 0.823 |  |  |
| Previous surgery | No | 1.08  (0.11–9.80) | 0.942 |  |  |
| Previous radiotherapy | Yes | 1.21  (0.45–3.21) | 0.702 |  |  |
| Number of previous chemotherapy regimens | 3- | 3.30  (0.41–26.4) | 0.260 |  |  |
| Number of organs with metastasis | 3- | 1.85  (0.39–8.69) | 0.431 |  |  |
| CD3 | Low | 1.01  (0.39–2.64) | 0.970 |  |  |
| CD8/Foxp3 | Low | 3.61  (1.22–10.6) | **0.0198** | 4.18  (1.26–13.9) | **0.0193** |
| TLS | Low | 7.05  (1.95–25.4) | **0.0029** | 7.81  (2.00–30.4) | **0.0030** |

Abbreviations: CI, confidence interval; HR, hazard ratio
