# Supplementary material for: Exploring predictive biomarkers of efficacy and survival with nivolumab treatment for unresectable/recurrent esophageal squamous cell carcinoma
Source: Esophagus. 2025 Apr 24;22(3):360–72. doi: 10.1007/s10388-025-01120-z (PMC12167336; doi:10.1007/s10388-025-01120-z)
Supplement: Supplementary file 9 — Supplementary file9 (DOCX 201 KB) [file 10388_2025_1120_MOESM9_ESM.docx]

Supplementary table S3. Univariate and multivariate analyses for non-response in endoscopic biopsies (n = 121)

| Variables | Category | Univariate analysis | | Multivariate　analysis | |
| --- | --- | --- | --- | --- | --- |
|  |  | HR  (95% CI) | *P* | HR  (95% CI) | *P* |
| Age (years) | ≤70 | 1.35  (0.45–3.98) | 0.586 |  |  |
| Sex | Female | 1.38  (0.43–4.34) | 0.581 |  |  |
| Performance status | 1–3 | 0.91  (0.31–2.60) | 0.859 |  |  |
| History of smoking | Yes | 0.72  (0.23–2.27) | 0.580 |  |  |
| Previous surgery | Yes | 1.73  (0.33–8.99) | 0.513 |  |  |
| Previous radiotherapy | Yes | 0.747  (0.25–2.16) | 0.591 |  |  |
| Number of previous chemotherapy regimens | 3- | 1.19  (0.31–4.64) | 0.797 |  |  |
| Number of organs with metastasis | 3- | 0.23  (0.03–1.88) | 0.174 |  |  |
| CD3 | Low | 1.31  (0.45–3.77) | 0.616 |  |  |
| CD8/Foxp3 | Low | 5.14  (1.38–19.1) | **0.0145** | 3.88  (0.96–15.5) | 0.0554 |
| CD8/CCR8 | Low | 3.42  (1.03–11.3) | **0.0433** | 2.12  (0.59–7.36) | 0.247 |

Abbreviations: CI, confidence interval; HR, hazard ratio
