# Supplementary material for: Exploring predictive biomarkers of efficacy and survival with nivolumab treatment for unresectable/recurrent esophageal squamous cell carcinoma
Source: Esophagus. 2025 Apr 24;22(3):360–72. doi: 10.1007/s10388-025-01120-z (PMC12167336; doi:10.1007/s10388-025-01120-z)
Supplement: Supplementary file 10 — Supplementary file10 (DOCX 201 KB) [file 10388_2025_1120_MOESM10_ESM.docx]

Supplementary table S4. Univariate and multivariate analyses for **PFS** in surgical specimens (n = 129)

| Variables | Category | Univariate analysis | | Multivariate　analysis | |
| --- | --- | --- | --- | --- | --- |
|  |  | HR  (95% CI) | *P* | HR  (95% CI) | *P* |
| Age (years) | ≤70 | 0.97  (0.67–1.42) | 0.906 |  |  |
| Sex | Female | 1.09  (0.69–1.71) | 0.707 |  |  |
| Performance status | 1–3 | 1.54  (1.06–2.26) | **0.0244** | 1.15  (0.77–1.72) | 0.494 |
| History of smoking | Yes | 1.03  (0.68–1.58) | 0.868 |  |  |
| Previous surgery | No | 1.31  (0.53–3.22) | 0.555 |  |  |
| Previous radiotherapy | Yes | 0.89  (0.58–1.39) | 0.630 |  |  |
| Number of previous chemotherapy regimens | 3- | 0.93  (0.535–1.65) | 0.828 |  |  |
| Number of organs with metastases | 3- | 1.52  (0.939–2.49) | 0.0872 | 1.48  (0.90–2.43) | 0.119 |
| CD3 | Low | 1.19  (0.81–1.74) | 0.368 |  |  |
| CD8/Foxp3 | Low | 1.71  (1.16–2.51) | **0.0066** | 1.88  (1.26–2.80) | **0.0019** |
| TLS | Low | 2.34  (1.58–3.45) | **<0.0001** | 2.48  (1.64–3.77) | **<0.0001** |

Abbreviations: CI, confidence interval; HR, hazard ratio
