# Supplementary material for: Exploring predictive biomarkers of efficacy and survival with nivolumab treatment for unresectable/recurrent esophageal squamous cell carcinoma
Source: Esophagus. 2025 Apr 24;22(3):360–72. doi: 10.1007/s10388-025-01120-z (PMC12167336; doi:10.1007/s10388-025-01120-z)
Supplement: Supplementary file 11 — Supplementary file11 (DOCX 201 KB) [file 10388_2025_1120_MOESM11_ESM.docx]

Supplementary table S5. Univariate and multivariate analyses for **PFS** in endoscopic biopsies (n = 121)

| Variables | Category | Univariate analysis | | Multivariate　analysis | |
| --- | --- | --- | --- | --- | --- |
|  |  | HR (95% CI) | *P* | HR (95% CI) | *P* |
| Age (years) | ≤70 | 0.96  (0.66–1.41) | 0.846 |  |  |
| Sex | Female | 1.10  (0.72–1.70) | 0.657 |  |  |
| Performance status | 1–3 | 1.27  (0.87–1.86) | 0.214 |  |  |
| History of smoking | Yes | 0.81  (0.53–1.26) | 0.364 |  |  |
| Previous surgery | No | 1.79  (0.90–3.59) | 0.0960 | 1.64  (0.82–3.29) | 0.158 |
| Previous radiotherapy | Yes | 0.99  (0.67–1.45) | 0.960 |  |  |
| Number of previous chemotherapy regimens | 3- | 0.85  (0.52–1.41) | 0.533 |  |  |
| Number of organs with metastasis | 3- | 1.73  (1.08–2.80) | **0.0288** | 1.50  (0.93–2.45) | 0.0962 |
| CD3 | Low | 0.79  (0.55–1.16) | 0.232 |  |  |
| CD8/Foxp3 | Low | 1.22  (0.84–1.78) | 0.296 |  |  |
| CD8/CCR8 | Low | 1.65  (1.12–2.41) | **0.0105** | 1.60  (1.08–2.40) | **0.0206** |

Abbreviations: CI, confidence interval; HR, hazard ratio
